# SAGA Complex Subunits in *Candida albicans* Differentially Regulate Filamentation, Invasiveness, and Biofilm Formation

**DOI:** 10.3389/fcimb.2022.764711

**Published:** 2022-03-08

**Authors:** Saima Rashid, Tuana Oliveira Correia-Mesquita, Pablo Godoy, Raha Parvizi Omran, Malcolm Whiteway

**Affiliations:** Department of Biology, Concordia University, Montreal, QC, Canada

**Keywords:** *Candida albicans*, filamentation, biofilm formation, invasiveness, stress response, macrophages, SAGA acetyltransferase complex

## Abstract

SAGA (Spt-Ada-Gcn5-acetyltransferase) is a highly conserved, multiprotein co-activator complex that consists of five distinct modules. It has two enzymatic functions, a histone acetyltransferase (HAT) and a deubiquitinase (DUB) and plays a central role in processes such as transcription initiation, elongation, protein stability, and telomere maintenance. We analyzed conditional and null mutants of the SAGA complex module components in the fungal pathogen *Candida albicans*; Ngg1, (the HAT module); Ubp8, (the DUB module); Tra1, (the recruitment module), Spt7, (the architecture module) and Spt8, (the TBP interaction unit), and assessed their roles in a variety of cellular processes. We observed that *spt7Δ/Δ* and *spt8Δ/Δ* strains have a filamentous phenotype, and both are highly invasive in yeast growing conditions as compared to the wild type, while *ngg1Δ/Δ* and *ubp8Δ/Δ* are in yeast-locked state and non-invasive in both YPD media and filamentous induced conditions compared to wild type. RNA-sequencing-based transcriptional profiling of SAGA mutants reveals upregulation of hyphal specific genes in *spt7Δ/Δ* and *spt8Δ/Δ* strains and downregulation of ergosterol metabolism pathway. As well, *spt7Δ/Δ* and *spt8Δ/Δ* confer susceptibility to antifungal drugs, to acidic and alkaline pH, to high temperature, and to osmotic, oxidative, cell wall, and DNA damage stresses, indicating that these proteins are important for genotoxic and cellular stress responses. Despite having similar morphological phenotypes (constitutively filamentous and invasive) *spt7* and *spt8* mutants displayed variation in nuclear distribution where *spt7Δ/Δ* cells were frequently binucleate and *spt8Δ/Δ* cells were consistently mononucleate. We also observed that *spt7Δ/Δ* and *spt8Δ/Δ* mutants were quickly engulfed by macrophages compared to *ngg1Δ/Δ* and *ubp8Δ/Δ* strains. All these findings suggest that the SAGA complex modules can have contrasting functions where loss of Spt7 or Spt8 enhances filamentation and invasiveness while loss of Ngg1 or Ubp8 blocks these processes.

## Introduction

*Candida albicans* is a widely distributed fungus and an important cause of hospital-acquired infections (Nobile and Johnson, 2015). In healthy individuals, it can form a part of normal microflora and asymptomatically colonize the skin, the mouth, and the gastrointestinal and reproductive tracts (Achkar and Fries, 2010; Ganguly and Mitchell, 2011). Nonetheless, when anatomical barriers are damaged due to changes in nutritional state, prolonged antibiotic use, diseases, or immunosuppressant therapy, these conditions can result in *Candida* overgrowth (Calderone and Fonzi, 2001). Overall, such *C. albicans* infections vary from superficial mucosal growth to life threatening systemic infections. In hospitals, *C. albicans* is the 3rd most common nosocomial pathogen isolated from patient blood cultures, while systemic *Candidiasis* can be associated with mortality rates of up to 50%, making *C. albicans* a significant medical issue (Wisplinghoff et al., 2004; Tournu and Van Dijck, 2012; Mathé and Van Dijck, 2013).

A distinctive hallmark of *C. albicans* is that it can switch among different morphological forms: budding yeast, pseudo-hyphae, and true hyphae, according to host cell responses and different environmental conditions (Staab et al., 1999; Gasch, 2007). The switching of *C. albicans* to different morphological states is closely associated with pathogenesis and biofilm formation (Jabra-Rizk etal., 2004; Ramage et al., 2005; Calderone and Clancy, 2012; Chauvel et al., 2012). This pathogen can also form dense biofilms on various biomaterials that are resistant to antifungal agents (Kojic and Darouiche, 2004). Despite the widespread studies undertaken in *C. albicans*, we still lack a clear, comprehensive understanding of the complexity of cellular functions and transcriptional regulation of this opportunistic fungus.

In eukaryotic cells, remodeling of chromatin structure is key for modulating gene expression because nucleosomes create a barrier for the binding of transactivating factors such as transcriptional factors (TFs) and RNA polymerases (Green, 2005). The SAGA (Spt-Ada-Gcn5-acetyltransferase) complex is a multi-protein co-activator complex that regulates numerous cellular processes through post-translational modifications (Baker and Grant, 2007). The SAGA complex is highly conserved among eukaryotes, from *Saccharomyces cerevisiae* to humans (Koutelou et al., 2010; Lavoie et al., 2010; Gurskiĭ et al., 2013; Srivastava et al., 2015). It was first identified in *S. cerevisiae* and is a large 1.8 MDa complex with 18–20 protein subunits. The complex was first characterized as a histone acetyltransferase (HAT) but also has an enzymatic role as a histone deubiquitinase (DUB) (Grant et al., 1997; Henry et al., 2003). It also has structural functions and is involved in recruiting the Tata Binding Protein (TBP) to gene promoters to modulate gene transcription (Belotserkovskaya et al., 2000; Warfield et al., 2004). In addition, it can be anchored to promoter regions to repress transcription (Belotserkovskaya et al., 2000).

SAGA is subdivided into five distinct functional modules based on their enzymatic or structural function—the HAT module (Gcn5, Ada2, Ngg1, Sgf29) responsible for Histone H3 acetylation (Brownell et al., 1996; Grant et al., 1997); the DUB module (Ubp8, Sus1, Sgf73, Sgf11) responsible for Histone H2B de-ubiquitination (Henry et al., 2003); the recruitment module (Tra1), which interacts directly with transcriptional activator domains of TFs, such as Gcn4 and Gal4 (Brown and Gow, 1999; Basso et al., 2019); the core structural module (Taf5, Taf6, Taf9, Taf10, Taf12, Ada1, Spt20, Spt7) (Grant et al., 1997); and the TBP interaction unit (Spt8, Spt3), which recruits the TATA-binding protein to the promoters in order to regulate transcription (Belotserkovskaya et al., 2000; Mohibullah and Hahn, 2008; Han et al., 2014). In *S. cerevisiae*, SAGA influences the gene expression of approximately 10% of the genome. Genes controlled by SAGA include stress-responsive genes implicated in challenges such as oxidative agents, high temperature, DNA damage, unfolded proteins and carbon or nitrogen starvation (Daniel and Grant, 2007). Besides transcriptional activation, SAGA is also required for transcription elongation, mRNA export, and DNA repair (Wery et al., 2004; Evangelista et al., 2018).

In *C. albicans*, it has been shown that the SAGA subunit Ada2 appears to have conserved role on oxidative stress and azole response compared to *S. cerevisiae*, as demonstrated by ChIP-Chip and ChIP-qPCR analysis (Sellam et al., 2009). Laprade et al. (2002) showed a switch of Spt3 from positive regulation of filamentation in the budding yeast to a negative role on the pathogen, and a similar switch was seen for Gcn5 (Chang et al., 2015). This functional rewiring in *C. albicans* and *S. cerevisiae* is fairly common between transcriptional regulators and has been described for many TFs, and might be a factor on the evolutionary differentiation that separated these two ascomycetes into a pathogen and a bread-making yeast from a common ancestor (Whiteway et al., 2015). Evolutionary rewiring happened at roughly similar rates (using years of divergence from a common ancestor as the denominator) in higher eukaryotes (Carvunis et al., 2015). Fungal ascomycete lineages that diverged 300 million years ago fall in this category where evolutionary wiring occurred at similar rates as established for the regulator Ndt80 in *S. cerevisiae* and *C. albicans* (Nocedal et al., 2017).

The SAGA complexes present at the promoter regions of *C. albicans* are found to be associated with oxidative stress, unfolded protein response, virulence, and azole-resistance genes (Askew et al., 2009). Unveiling the role of the different modules of this complex will provide better understanding of the puzzling regulatory circuitry of SAGA complex in *C. albicans* common to most environmental cues. In this work, we investigated conditional and null mutants of components of the SAGA complex modules; Ngg1 of the HAT module, Ubp8 of the DUB module, Tra1 of the recruitment module, Spt7 of the architecture module, and Spt8 of the TBP interaction unit to assess their role in processes such as filamentation, invasiveness, and biofilm formation. It appears Tra1 is essential, like its orthologue in *S. cerevisiae*, as we failed to get the homozygous deletion of this gene. We identified that SAGA complex modules can work in an antagonistic manner. We showed that Spt7 and Spt8 have an important regulatory role in response to cell-wall, osmotic, temperature and drug stresses while as Ngg1and Ubp8 has a regulatory role in response to temperature. All these outcomes imply that Spt7 and Spt8 are indispensable for regulation of characteristics such as cell morphology, genotoxic and cellular stress responses and responses to antifungal drugs.

## Materials and Methods

### Strains and Oligonucleotides

The conditional repressed mutants from the SAGA complex used in this study were obtained from the GRACE™ collection (Roemer et al., 2003). The parental CaSS1 strain and all the GRACE library strains have transactivation fusion protein consisting of the *E. coli* tetR binding domain, linked with *URA3* module and the *S. cerevisiae GAL4* activation domain. Conditional repression of individual mutant strains can be achieved by growing the strains in the YPD media supplemented with 100 μg/ml tetracycline, and null mutant can be obtained by growing them on 5 Fluoorotic acid (5-FOA) containing medium to detect loss of the *URA3*-linked module (Roemer et al., 2003). The starting strain used for the construction of the SAGA deletion mutants was SN148 (Nobile and Johnson, 2005). The knock-out mutants of *SPT7*, *SPT8*, *NGG1*, and *UBP8* were built using the CRISPR/Cas9 method as described (Vyas et al., 2015). The *TRA1* mutant was attempted by classic homologous recombination replacement using *HIS1* and *URA3* as selectable markers. **Table S1** contains the genotype descriptions for all the mutants constructed and strains mentioned. PCR and DNA sequencing were used to confirm all mutants mentioned in this work. Oligos used to obtain and confirm the knock-out mutations are listed in **Table S2**.

### Media

Yeast colonies were grown in yeast-peptone-dextrose YPD media (1% w/v yeast extract, 2% w/v Bacto peptone, 2% w/v dextrose, 100 mg/L uridine with the addition of 2% w/v agar for solid medium) for 48 h at 30°C. Yeast cells were cultured overnight from fresh single colonies and diluted in YPD liquid media to a starting OD_600_ of 0.2, and cultured for 4 h for normal growing strains and 6 h for slow growing strains at 30°C, 220 rpm. Hyphal colonies were induced in 10% serum supplemented YPD media plates containing 2% agar and in Spider media (1% Difco nutrient broth, 1% mannitol, 0.2% dibasic potassium phosphate, pH 7.2) plates containing 2% agar for 5 days at 37°C. Hyphal cells were induced from overnight YPD cultures diluted in 10% serum supplemented YPD and Spider media from a starting OD_600_ of 0.2 and incubated for 4 h for normal growing strains and 6 h for slow growing strains at 37°C respectively, 220 rpm. All assays performed with the conditional repressed mutants were supplemented with 100 µg/ml tetracycline.

### Microscopy

For cell morphology, overnight cultures were grown in non-inducing media—YPD at 30°C and inducing media—Spider media and 10% fetal calf serum and were subjected to phase differential interference contrast microscopy. Cell morphology was assessed under ×100 magnification. A total of 1,000 cells were counted and divided into three morphological categories: yeast, pseudo-hyphae, and hyphae. Four biological replicates were made for each mutant from the SAGA complex. The results were analyzed, and the graphs were made using Microsoft. For nuclear segregation analysis, DAPI staining of live cells were performed without permeabilization. Overnight cultures were resuspended at a starting optical density 600 nm (OD_600_) = 0.2 in YPD medium (1% w/v yeast extract, 2% w/v Bacto peptone, 2% w/v dextrose, 80 mg/L uridine, and incubated for 4 to 6 h (until successful completion of first cellular division of both wild type and mutant strains). To visualize DNA, cells were washed twice with 1× PBS followed by the addition of 3 μg/ml DAPI (Sigma-Aldrich) into each tube. To visualize cell membrane and chitin distribution, Calcofluor staining (1.5 μg/ml) was performed using similar strategy. Cells were examined by DIC and fluorescent microscopy at ×100 magnification using a Leica DM 6000 microscope (Leica Microsystems Canada, Richmond Hill, ON, Canada) equipped with a Hamamatsu-ORCA ER camera (Hamamatsu Photonics, Hamamatsu City, Japan) and the HCX PLFLUO TAR 100× NA 1.30–0.6 oil objectives. Differential interference contrast optics or epifluorescence with DAPI (460 nm) filters were utilized. Images were captured with Volocity software (Improvision, Perkin-Elmer, Waltham, MA) and images were analyzed using ImageJ/Fiji software.

### Phenotypic Sensitivity

To test sensitivity phenotypes, mutant strains from the SAGA complex were subjected to different stress conditions. The strains were inoculated from single colonies in 5 ml YPD and incubated at 30°C, overnight, and were diluted to OD_600_ 0.2. The starting dilution was used in a subsequent 1:10 serial dilution and 3 µl of each dilution were spotted onto the stress plates containing YPD agar media supplemented with menadione (0.15 mM) and hydrogen peroxide (7.5 mM) were used for oxidative stress assays; methyl methane sulfonate (MMS, 0.01 v/v), hydroxyurea (15 mM) were used in DNA damage stress assays; and Congo red (200 µg/ml) and antifungal Caspofungin (0.75 µg/ml) were used to test the mutants for cell wall stress; dithiothreitol (DTT 30 mM) provided ER stress by forcing the accumulation of unfolded proteins; NaCl (1.5 M), CaCl_2_ (400 mM) and glycerol (250 mM) were used for osmotic stress; fluconazole (10 µg/ml), hygromycin B (100 µg/ml), and anidulafungin (0.25 µg/ml) treatments were used to trigger antifungal drug responses. All YPD plates were incubated at 30°C for 4 days except for Caspofungin (200 μg/ml) containing plates that were incubated for 7 days at 30°C. To test for the ability to grow under temperature stress of mutants, growth on YPD agar plates at 37 and 42°C was tested. YPD agar plates at pH 8.3 and pH 5 were used to test the mutants for response to alkaline and acidic stresses with incubation at 30°C for 48 h.

### Invasiveness Assay

Overnight cultures from fresh single colonies were grown in liquid YPD at 30°C and 220 rpm and diluted to an OD_600_ of 0.1.5 µl samples were spotted on YPD agar plates and Spider media plates and incubated at 30 and 37°C for 120 h. The resulting colonies were then washed gently under running water for 15 s to remove the non-adherent surface cells, and the invasiveness of the samples was observed. The colonies that remained on the plates after washing were considered invasive and those washed away were counted non-invasive. Two biological replicates were prepared for each sample. The plates were scanned before and after washing at 600 dots per inch (dpi) using an Epson Perfection v500 photo scanner.

### Biofilm Assay

The strains were inoculated in 5 ml liquid Spider media and incubated at 24°C for 24 h. Approximately 4 × 10^7^ cells of each sample were added to 1 ml of Lee’s media in a 24-well flat-bottom plate. The media was discarded, and the biofilms were washed three times with 1 ml DPBS buffer. The plates were allowed to dry, and the biofilms were stained with 325 µl of 0.4% crystal violet for 45 min. The staining solution was washed 3 times with 1 ml of sterile Milli-Q water and allowed to dry. The biofilms were de-stained with 500 µl 95% ethanol. The amount of biofilm was measured based on the absorbance at 595 nm. For each sample, three biological replicates were prepared. Results were analyzed and graphs made with GraphPad Prism (version 6.0).

### Macrophage Engulfment Assay

The RAW 264.7 murine macrophage cell line was kindly provided by Dr. Albert Descoteaux (INRS-Armand-Frappier, Laval, QC, Canada). Macrophages were cultured in DMEM medium supplemented with 10% FBS, penicillin/streptomycin and HEPES. Macrophages were seeded at 2 × 10^5^ and grown for 48 h at 37°C and 5% CO2. Once cells reached 80% confluency, macrophages were collected with Trypsin-EDTA and centrifuged for 10 min at 10,000× rpm, at room temperature. Then cells were stained with trypan blue and counted. An aliquot of 1.2 × 106 cells/ml was prepared for further macrophage engulfment assays.

Knock-out mutants *ngg1Δ/Δ*, *spt7Δ/Δ*, *spt8Δ/Δ*, and *ubp8Δ/Δ*, derived from the parental strain SN148 were grown overnight in 5 ml YPD medium at 37°C, 220× rpm shaking incubation. An aliquot of each mutant and the parental strains was taken to grow again for 3 h prior to assay. An aliquot of each SAGA mutant and the parental strain were spun down, the media removed and cell pellet washed 3 times with PBS (phosphate buffer saline) and adjusted to a final fungal cell concentration of 1 × 10^8^ cells/ml. Fungal cells were then stained with 50 mg/ml of Calcofluor White (CFW, Sigma), and incubated for 10 min at RT, then washed three times in PBS and finally a 1/100 dilution in PBS was prepared for each strain in the assay.

Both macrophages and fungal cells for each strain were mixed in a ratio 1:10 (Candida cell: macrophage) in a well of a 96-well plate and then visualized in a high-content screening microscope ImageXpress XSL wide-field (Molecular Devices). The plate was placed in a chamber equilibrated at 37°C and 5% CO_2_. Images were captured at ×40 objective magnification on two channels (transmitted light and DAPI), at time point 0 and then every 5 min for 4 h total running time. After, for every SAGA strain and macrophage images, a time-lapse video was generated, using the MetaXpress high content imaging acquisition and analysis software (Version 6.1.1, Molecular Devices). Fungal cells (budding or filament forms) engulfed by macrophages, were counted for every time-point, and normalized to the ratio: [number of fungal cells at time point 0/number of fungal cells for every time point] × 100. Macrophage engulfment kinetic curves, histograms, and statistical analysis were analyzed with GraphPad Prism (Version 6.0).

### RNAseq Analysis

The deletion mutant strains and control SN148 cultures were grown in YPD media from a starting OD_600_ of 0.1 and the cultures were allowed to grow at 30°C, 220 rpm until OD_600_ 1.0. Total RNA was extracted using the Qiagen RNeasy minikit. The quality of RNA was assessed *via* Agilent 2100 Bioanalyzer using the Agilent RNA 6000 Nano kit. The RNAseq was performed by the McGill University and the Genome Quebec Innovation Centre using an Illumina MiSeq. Raw reads were pre-processed with the sequence-grooming tool cutadapt version 0.4.1 (Martin, 2011) with the following quality trimming and filtering parameters (`–phred33 –length 36 -q 5 –stringency 1 -e 0.1`). Each set of paired ends read was mapped against the *C. albicans* SC5314 haplotype A, version A22 downloaded from the Candida Genome Database (CGD) (http://www.candidagenome.org/) using HISAT2 version 2.0.4. SAMtools was then used to sort and convert SAM files. The read alignments and *C. albicans* SC5314 genome annotation were provided as input into 13 StringTie v1.3.3 (Pertea et al., 2015), which returned gene abundances for each sample.

### Statistical Analysis

Data are presented as means ± standard errors of the means from separate experiments and were compared using one-way analysis of variance (ANOVA) and student’s *t*-test. The level of significance was set at a P-value of <0.05. All statistical analyses were performed using GraphPad Prism (version 6) statistical software (GraphPad Software, San Diego, CA) and Microsoft Excel.

## Results

### SAGA Mutants Can Have Opposing Consequences for Filamentation and Invasiveness

To investigate mutants belonging to different modules of the SAGA complex in *C. albicans*, we initially made use of conditionally repressed mutants from the GRACE™ library (Roemer et al., 2003), namely, *TRA1*—*ORF19.139* (recruitment module), *NGG1*—*ORF19.3023* (HAT module), *SPT7*—*ORF19.7572* (architecture unit), and *SPT8*—*ORF19.4312* (TBP-associated unit); these genotypes are described in **Table S1**. We assessed the role of conditional SAGA mutants in filamentation and invasiveness in presence or absence of tetracycline (100 μg/ml). We found that conditional mutants *spt7* and *spt8* were filamentous and invasive in both filamentous inducing and non-inducing conditions while *tra1Δ* and *ngg1Δ* were in a yeast-locked state compared to wild type. As well, similar to *spt3Δ/Δ* and *spt20Δ/Δ* deleted strains in *C. albicans* (Laprade et al., 2002; Wang et al., 2020), we found cells of the *spt7* and *spt8* conditional mutants did not separate properly during cell division and appeared clumped together. Also, the *spt7* and *spt8* repressed mutants formed wrinkled colonies in both inducing and non-inducing conditions—indication of filamentous cells, while the *ngg1* and *tra1* repressed mutants formed smooth colonies even in the hyphal-inducing conditions of either 10% fetal calf serum (FCS) or Spider media at 37°C for 5 days when compared to the wild type CaSS1 strain (data not shown).

However, there are numerous issues associated with repression of gene transcription—the possibility of promoter leakage, the fact that the non-repressed mutants might have increased expression of routinely low expressed genes and therefore activate biological processes that in a wild type background would not be active, and ultimately the necessity of addition of tetracycline or doxycycline, which are iron chelators, and might act as a source of stress to the mutant (Samaranayake and Hanes, 2011; Fiori and Van Dijck, 2012). Also, the GRACE™ library is not comprehensive, as some SAGA modules (like the DUB module) do not have representatives in the collection. Therefore, we created null mutants for Spt7 from the architecture unit, Spt8 from the TBP-binding module, Ngg1 from the HAT-module, and Ubp8 from the de-ubiquitination module; these genotypes are described in **Table S1**. We failed to create a Tra1 null mutant strain despite repeated attempts. However, the Tra1 GRACE library strain was viable under repressing conditions, and when this library strain was grown on 5-FOA media to create a null mutant by removing the trans-activator tetR binding domain cassette linked with the *URA*3 gene, we were able to get colonies on 5-FOA agar media. The removal of the trans-activator domain was confirmed through PCR. These observations had suggested that Tra1 may not be essential in *C. albicans*; this would be unprecedented, because the Tra1 function is essential in all other organisms investigated, and there is no evidence for a duplicated gene in *C. albicans*. However, when we attempted to remove the FATC domain (C-terminal domain) of Tra1, which plays an important role in cellular viability as part of its orthologue in *S. cerevisiae* (Hoke et al., 2010), we failed to get homozygous deletion of the domain. This would suggest that Tra1 is in fact an essential protein in the SAGA complex, as previously found by *in vivo* transposon mutagenesis and machine learning analysis in a stable haploid isolate of *C. albicans* (Segal et al., 2018); but because of the complexity introduced by the GRACE strain phenotype this point needs further confirmation.

**Figure 1:**
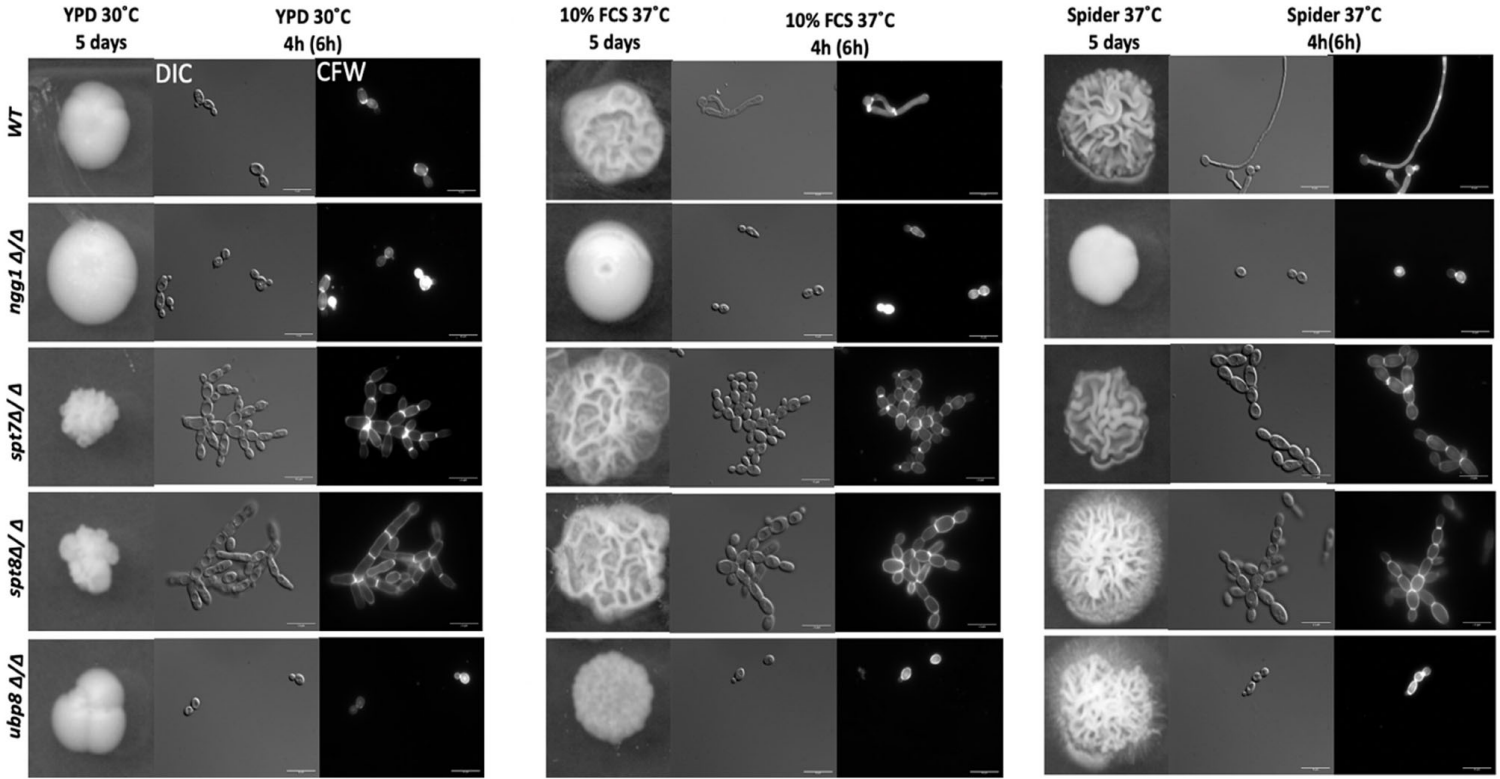
Colony and cellular morphology of SAGA mutants in *C. albicans*. 1:10 serial dilution of overnight culture of mutants were spotted on to yeast-growing conditions—YPD agar and hyphae-inducing conditions—10% fetal calf serum media (FCS) and Spider media plates. The colony morphology was assessed after 5 days of incubation. Cells from liquid media were inoculated at starting OD_600_ of 0.2 and grown in liquid YPD, 10% FCS supplemented YPD and Spider medium, at 220 rpm, and 30°C or 37°C for 4 h for normal growing strains (WT, *ngg1Δ/Δ* and *ubp8Δ/Δ*) and 6 h for slow growing strains (*spt7Δ/Δ* and *spt8Δ/Δ)*. The cells were washed with 1× PBS twice and stained with 2 µg/ml calcofluor white (CFW). The cells were observed with the Leica DM6000 microscope at ×100 magnification-DIC (Differential Interference Contrast). Scale bar = 15 µm. *spt7Δ/Δ* and *spt8Δ/Δ* appear more hyphal compared to control and are mostly in pseudo-hyphal state in inducing and non- inducing media whereas *ngg1Δ/Δ* and *ubp8Δ/Δ* appear in yeast locked state. The control switches its morphology upon changed conditions while the SAGA mutants remain in their initial states upon induction.

We assessed the colony morphology of SAGA mutants in normal yeast growing conditions (YPD) and both rich and starvation hyphal-inducing conditions specifically, 10% fetal calf serum (FCS) and mannitol-based Spider media at 37°C. As shown in **Figure 1**, under yeast-growing conditions, both *spt7Δ/Δ* and *spt8Δ/Δ* null mutants generate wrinkled, crenulated colonies, and this wrinkled phenotype intensified when we induced hyphae by growth on 10% FCS or Spider media. The wrinkled and crenulated colonies indicate the presence of filamentous cells. These mutant cells are very filamentous when grown in liquid YPD media, showing a mix of hyphae and pseudo-hyphae, but mainly pseudo-hyphae with branched filaments. The same phenotype was observed when the mutants were induced to form hyphae in 10% FCS or Spider media at 37°C for 4 h. The *spt8Δ/Δ* phenotype is consistent with that of the hyper-filamentous Spt3 deleted strain in *C. albicans*, which removes a subunit of the same module (Laprade et al., 2002). Similar to *spt3Δ/Δ* and *spt20Δ/Δ* deleted strains in *C. albicans* (Laprade et al., 2002; Desai and Mitchell, 2015), we also found *spt7* and *spt8* mutant cells didn’t separate properly during cell division and appeared clumped together. In contrast, the colonies of the deleted *ngg1* strain were smooth on either hyphae-inducing medium. The cells also appear mainly yeast or pseudo-hyphal in liquid media after hyphae induction at 37°C in both hyphal inducing conditions. The *ubp8* null from the deubiquitylation module shows classic yeast morphology in both non-inducing-YPD media and hyphae-inducing media-10% FCS or Spider media at 37°C (**Figure 1**). Overall, these results indicate that filamentation of *spt7Δ/Δ* and *spt8Δ/Δ* under both inducing and non-inducing conditions might influence a common, core component of the cellular machinery that plays a role hyphal formation, most likely a component downstream of multiple different signaling pathways.

**Figure 2:**
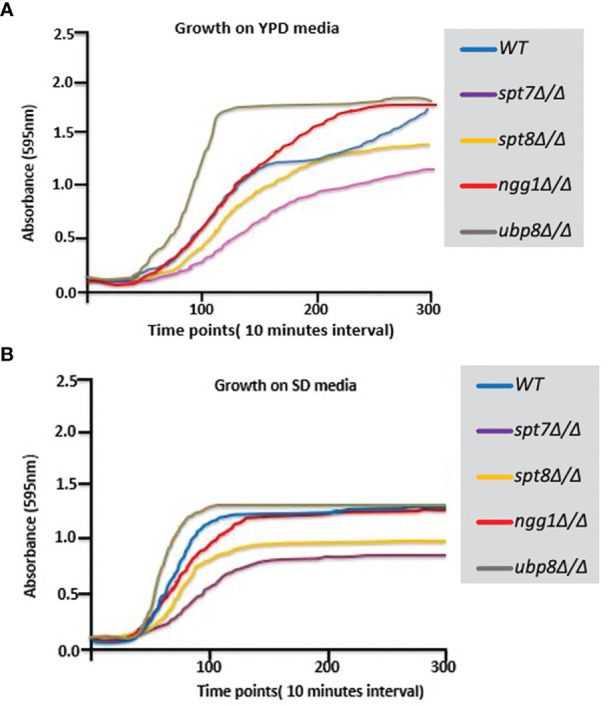
Growth curves of SAGA mutants. **(A, B)** Graphs showing growth rates of mutants on YPD and SD media. Growth rate of each strain was assessed using Sunrise™ TECAN plate reader over a period of 5 days by following the growth of 7 biological replicates from a starting OD_600_ 0.001 in 200 µl YPD in 96 well plates at 30°C. Results were analyzed, and graphs were plotted. *spt7Δ/Δ* and *spt8Δ/Δ* strains grew slowly compared to WT; *ngg1Δ/Δ* and *ubp8Δ/Δ* grew normally compared to WT.

We have extended the assessment of the phenotypes of these 4 non-essential SAGA module components. *spt7Δ/Δ* and *spt8Δ/Δ* deleted mutants grow slower compared to the background strain (SN148), so their growth was observed in rich YPD and SD media at 30°C at different time intervals. As shown in **Figures 2A, B**, the *spt7Δ/Δ* and *spt8Δ/Δ* mutant strains grew considerably slower compared to the wild type during the first 30 h of growth in YPD at 30°C. The *ubp8Δ/Δ* mutant strain has the opposite behavior, growing slightly faster than the wild type SN148 strain in rich media. There is also description of the hyper-filamentous, slow-growing *spt3Δ/Δ* deleted mutant in *C. albicans* (Laprade et al., 2002) similar to the *spt8Δ/Δ* deleted mutant affecting another subunit of same SAGA module. This evidence suggests a role of negative regulation on filamentation of the TBP-interaction unit. This supports our observation that Spt7 and Spt8 act in the negative regulation of filamentation whereas Ngg1 and Ubp8 appear to function in positive regulation of hyphal development.

**Figure 3:**
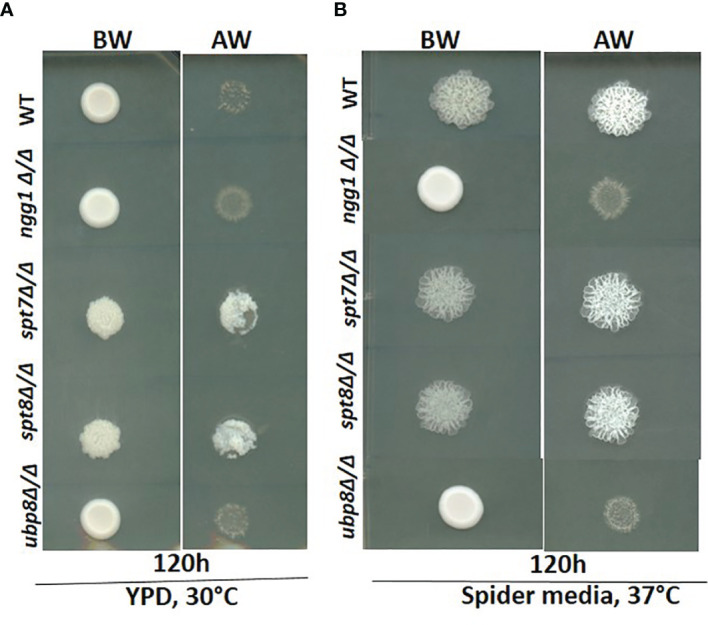
Invasiveness of SAGA mutants in C. albicans. Overnight cultures from fresh single colonies were grown in liquid YPD at 30°C and 220 rpm and diluted to an OD600 of 1. 3μL samples were spotted on YPD agar plates and Spider media plates and incubated at 30°C and 37°C for 120h. The resulting colonies were then washed gently under running water for 15 seconds to remove the non-adherent surface cells, and the invasiveness of the samples was observed. **(A, B)** The spt7Δ/Δ and spt8Δ/Δ knock-out mutants were found to be invasive. However, ngg1Δ/Δ and ubp8Δ/Δ were non-invasive in both conditions compared to its control. BW-Before wash; AW-After Wash.

Since invasiveness in *C. albicans* is often associated with virulence, we tested the null strains for invasion in a plate-washing assay. An overnight grown culture was spotted onto YPD agar at 30°C and Spider media at 37°C respectively and incubated for 120 h followed by washing with a stream of Milli-Q water for 15 s. As shown in **Figure 3**, after 120 h incubation the *spt7Δ/Δ* and *spt8Δ/Δ* mutants were more invasive than the control in both yeast-growing and filamentous conditions at 30 and 37°C whereas *ngg1Δ/Δ* and *ubp8Δ/Δ* were non-invasive like the wildtype in normal yeast growing conditions (**Figure 3**). The Spt7 and Spt8 knock out mutants were the most constitutively invasive, consistent with their hyper-filamentous phenotype. The plate-washing assay on YPD media at 30°C showed the invasive phenotype when there was no inducing signal present, suggesting the *spt7Δ/Δ* and *spt8Δ/Δ* deleted mutants are constitutively activated. The spots for *spt7Δ/Δ* and *spt8Δ/Δ* were considerably more invasive compared to non-invasive conditions (**Figure 3**), indicating a strong role of Spt7 and Spt8 on the negative regulation of invasion while Ngg1 and Ubp8 act positively as does Gcn5, a member of the HAT module like Ngg1 (Chang et al., 2015).

### Cellular Characteristics of SAGA Mutants

To study the cellular phenotypes of SAGA mutants, we grew the mutants in both non-inducing (YPD medium) and filamentous inducing liquid media (Spider media and 10% FCS) at 30 and 37°C respectively. We found that *spt7Δ/Δ* and *spt8Δ/Δ* cells displayed abnormal phenotypes in both filamentous inducing and non-filamentous inducing media where individual mutant cells were morphologically abnormal ranging from enlarged and elongated yeast-like cells to pseudo-hyphal cells. The *ngg1Δ/Δ* and *ubp8Δ/Δ* cells were in an enlarged yeast-locked state compared to their isogenic wild type. The major percentage of cells in the *spt7* and *spt8* deleted strains exhibit a pseudo hyphal form in both inducing and non-inducing media compared to wild type. By contrast, the *ngg1* and *ubp8* deleted strains display a most cells in the yeast form in all inducing and non-inducing media (**Tables 1.1**–**1.3**). We further measured the cell size of the SAGA mutants in log phase yeast growth conditions. The wild-type yeast cells were in range of 6.4–23.8 μm^2^. The yeast-locked cells of *ngg1* and *ubp8* deleted mutants were larger, in the range of 11.2–59.8 and 11.1–27.1 μm^2^ respectively. Furthermore, the *spt7* and *spt8* deleted cells grew as clusters and were four to five times the size of the wild type cells. Thus, the deletion of SAGA subunits has a significant impact on the cellular morphology of *C. albicans*.

**Table 1.1:** Table showing percent of total cell count of SAGA module subunits in YPD media.

Strains	Yeast	Hyphal	Pseudo-hyphal
**WT**	100% (± 1.5%)	0% (± 0%)	0% (± 0%)
***ngg1Δ/Δ***	92% (± 1.5%)	0% (± 0%)	8% (± 1%)
***spt7 Δ/Δ***	5% (± 0.5%)	7% (± 1%)	88% (± 2.5%)
***spt8Δ/Δ***	6% (± 0.5%)	2% (± 1%)	92% (± 2.5%)
***ubp8Δ/Δ***	91% (± 2.5%)	0% (± 0%)	9% (± 1.75%)

**Table 1.2:** Table showing percent of total cell count of SAGA module subunits in spider media.

Strains	Yeast	Hyphal	Pseudo-hyphal
**WT**	10% (± 1.5%)	71% (± 3%)	19% (± 2%)
***ngg1Δ/Δ***	91% (± 1%)	0% (± 0%)	9% (± 1.5%)
***spt7 Δ/Δ***	4% (± 0.5%)	7% (± 1%)	89% (± 3%)
***spt8Δ/Δ***	4% (± 0.5%)	4% (± 0.5%)	92% (± 2.5%)
***ubp8Δ/Δ***	87% (± 2%)	0% (± 0%)	13% (± 1.5%)

**Table 1.3:** Table showing percent of total cell count of SAGA module subunits in 10% Fetal Calf Serum.

Strains	Yeast	Hyphal	Pseudo-hyphal
**WT**	12% (± 1%)	69% (± 2.5%)	19% (± 1.5%)
***ngg1Δ/Δ***	89% (± 1%)	0% (± 0%)	11% (± 1.75%)
***spt7 Δ/Δ***	2% (± 0.5%)	5% (± 0.5%)	93% (± 2.5%)
***spt8Δ/Δ***	5% (± 0.5%)	4% (± 0.75%)	91% (± 1.5%)
***ubp8Δ/Δ***	86% (± 3%)	0% (± 0%)	14% (± 2%)

Standard deviations are shown in brackets.

The SAGA *spt7* and *spt8* deletion mutants grew slowly on plates and liquid media. We measured the cell density of the SAGA mutants after 24 h incubation by direct hemocytometer counts. From a starting count of 1 × 10^7^ cells/ml—the *spt7* and *spt8* deleted mutants reached a density of 20 × 10^7^ and 25 × 10^7^ cells/ml respectively after a 24-hour incubation at 30°C in liquid YPD media, while the *ngg1* and *ubp8* deleted cells reached 75 × 10^7^ and 91 × 10^7^ cells/ml and the wild type reached 84 × 10^7^ cells/ml. These results highlight the impact of Spt7 and Spt8 on the growth of *C albicans*.

### Spt7 and Spt8 Mutant Strains Displayed Cell Cycle Related Defects

**Figure 4:**
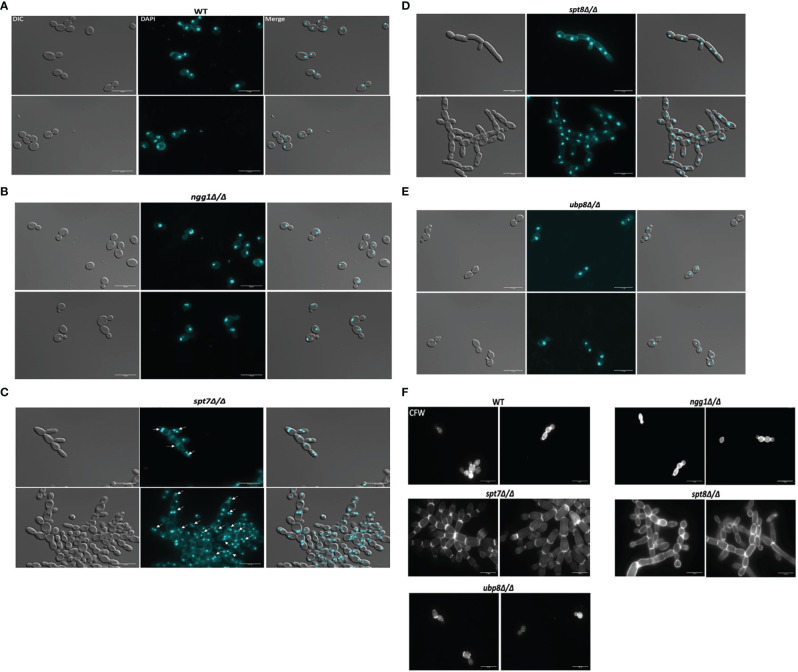
Staining of the SAGA mutants with DAPI and calcofluor white. Cultures were allowed to grow for 4 h for wild type, *ngg1Δ/Δ*, *ubp8Δ/Δ* and for 6 h for *spt7Δ/Δ* and *spt8Δ/Δ* under yeast growth conditions. Cells were washed twice with 1× PBS and then stained with 3 μg/ml DAPI or with 1.5 μg/ml calcofluor and mounted on slides. Individual cells were examined under ×100 magnification using LEICA DM 6000 microscope, scale bar 15 μm. **(A–E)** All SAGA mutant cells showed normal nuclear segregation with each individual cell carrying single nuclei except **(C)** *spt7Δ/Δ* mutants were frequently binucleate (shown by white arrow heads) compared to its wild type. **(F)** Calcofluor white (CFW) stained cells displayed even chitin distribution and highly noticeable septa in all SAGA mutants similar to the wild type strain.

To study the effects of SAGA mutants on nuclear segregation, mutant cells of each SAGA subunit (n = 210) and wild type cells (n = 210) were stained with DAPI and observed under microscope. The *spt7* and *spt8* deleted mutants showed similar phenotypes of cell clumping and difficulties in separation, so we investigated the patterns of nuclear distribution. Intriguingly, they showed considerable differences in their nuclear distribution, as 34% of *spt7Δ/Δ* cells were binucleate (n = 72), 18% have diffuse nuclei (n = 37), and 48% were mononucleate (n = 100), whereas 95% of the *spt8Δ/Δ* deleted cells were mononucleated (n = 199). Both *ngg1Δ/Δ* and *ubp8Δ/Δ* mutant cells showed normal patterns of yeast cell morphogenesis where 94% large, budded cells have two nuclei, one in each bud cell and mother cell (n = 98), while 90% (n = 94) of small, budded cells have nuclei at the junction of bud neck and mother cell similar to wild type strain where 90% (n = 94) of large budded cells have 2 nuclei, one in each bud and mother cell while 92% (n = 97) of small budded cells have nuclei at the junction of the bud neck and mother cells (**Figures 4A–E**). These results suggest that in the filamentous phenotypes of SAGA mutants the cells might be in late S/G2 phase or defective/late M phase, potentially due to issues in DNA repair machinery which resulted in abnormal nuclear content and an increased cell size. For nuclear segregation between mother and daughter cells, septal ring formation is required (Berman, 2006). Further we tested the SAGA deleted mutants for the chitin composition of cell wall and septa using calcofluor staining. All the strains showed uniform chitin distribution in their cell walls and at septal junctions having prominent and distinct septa like the wild type (**Figure 4F**). All these morphological defects in *spt7* and *spt8* deleted strains (slow growth, enlarged cell size, filamentation, and abnormal nuclear segregation) indicate that Spt7 and Spt8 are needed for normal cellular physiology in *C. albicans*.

### SAGA Complex Subunits Appear to Differentially Influence Biofilm Regulation

**Figure 5:**
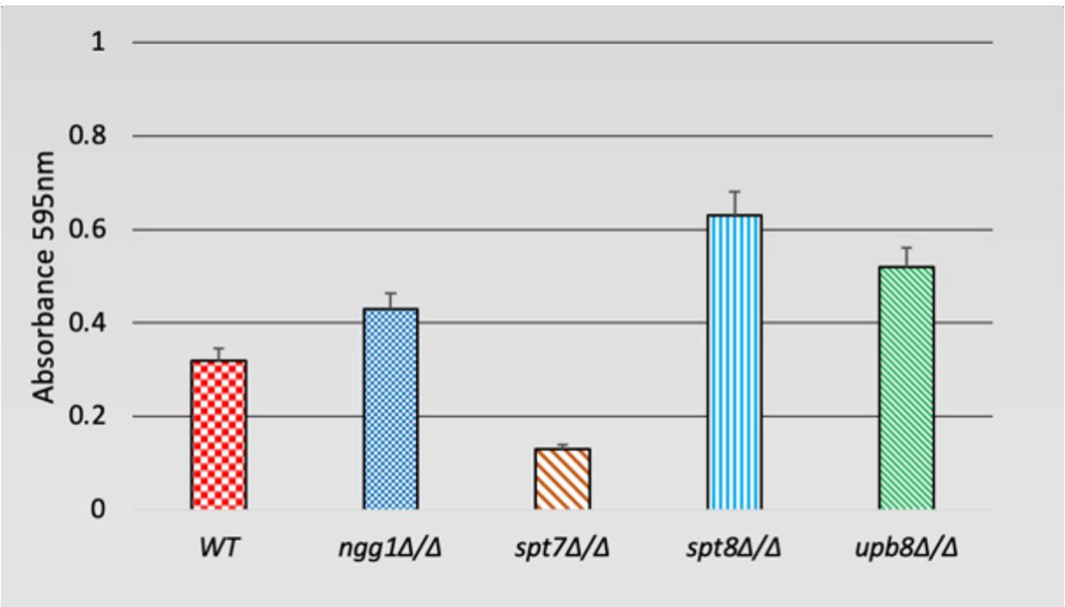
Biofilm formation of SAGA mutants in *C. albicans*. Quantification of biofilm formation in de-staining solution. Deleted mutants of SAGA complex were tested for biofilm formation in Lee’s medium after 48 h. It appears *spt8Δ/Δ* enhances biofilm formation whereas *spt7Δ/Δ* showed a decrease in biofilm formation compared to its control. Error bars indicates standard deviation.

Filamentation is often associated with the ability to form biofilms, which is considered an important factor for hospital-acquired infections (Chandra et al., 2001; Kojic and Darouiche, 2004; Desai and Mitchell, 2015; Tsui et al., 2016). We tested the deleted mutants for biofilm formation in Lee’s medium after 48 h of growth. While the *spt7Δ/Δ* and *spt8Δ/Δ* mutants shared many phenotypic similarities, it appears that the *spt8Δ/Δ* strain showed a somewhat increased biofilm formation compared to WT, while the *spt7Δ/Δ* strain showed decreased biofilm formation. We did not observe any significant difference in the *ngg1* and *ubp8* deleted strains tested in the regular biofilm induction (**Figure 5**).

### Oxidative, Osmotic, Cell Wall and Temperature Stresses in *C. albicans* are Differentially Influenced by SAGA Sub-Modules

**Figure 6:**
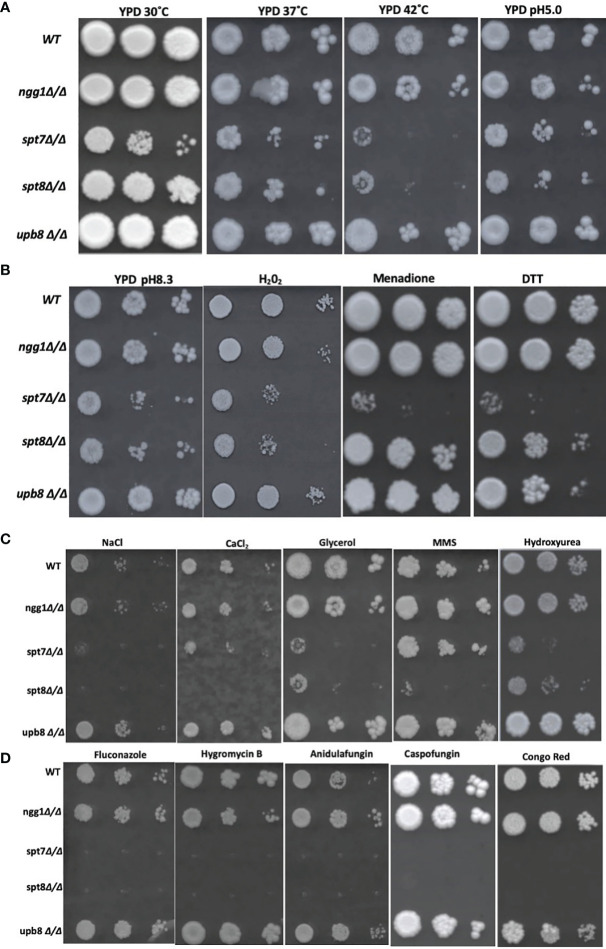
Genotoxic and cellular stress assay and antifungal drugs response of SAGA mutants in *C*. *albicans*. **(A, B)** 1:10 serial dilution of Overnight cultures grown in yeast growth conditions were spotted starting with OD of 0.2 onto YPD agar plates containing different chemicals and were incubated at 30°C for 4 days except for Caspofungin (7 days) strains response to heat (37 and 42°C) and alkaline medium (pH 8.3). Strains were subjected to acidic stress (pH 5.0), oxidative stress (Hydrogen peroxide 7.5 mM, 0.15 mM Menadione) and ER stress (DTT, Dithiothreitol (30 mM). **(C)** Strains were subjected to osmotic stress (NaCl 1.5 mM, CaCl2 400 mM, glycerol 250 mM) and genotoxic stress of Methyl Methane Sulfonate (0.01% v/v), Hydroxy Urea (15 mM) and Hydrogen peroxide (7.5 mM), **(D)** to determine resistance to different cell membrane damaging drugs, fluconazole (10 μg/ml), hygromycin B (100 μg/ml), anidulafungin (0.25 μg/ml), and cell wall stress caused by Caspofungin (0.75 μg/ml) and Congo red (200 μg/ml). Experiment was repeated 3 times for each sample.

Environmental stresses are often associated with SAGA complex influence in *S. cerevisiae* (Huisinga and Pugh, 2004), so we investigated the consequences of the subunit mutations on response to a variety of stress conditions. SAGA knock-out mutants led to sensitivity to high temperature stress in *S. cerevisiae* as proved for *ngg1* and *ubp8* mutants through classical genetics and *spt7* and *spt8* mutants through large scale survey (Amerik et al., 2000; Sinha et al., 2008; Ruiz-Roig et al., 2010). We did spot assays on YPD agar media and plates were incubated at 37 and 42°C to assess the consequences of SAGA subunit loss in *C. albicans*. We found *ngg1Δ/Δ* and *ubp8Δ/Δ* were resistant to 42°C incubation. However, *spt7Δ/Δ* and *spt8Δ/Δ* were sensitive at 37°C compared to wildtype (**Figure 6A**). This result supports the idea that significant functional rewiring has taken place within this complex between the two species. In *S. cerevisiae*, *ngg1* and *ubp8* mutants are temperature sensitive whereas in *C. albicans ngg1* and *ubp8* mutants are resistant to high temperature. This suggests that the elements that are positively influencing temperature stress response in one organism have a negative influence on the same stress in a closely related fungus. This further highlight that Spt7 and Spt8 are required to cope up with increased temperature in *C. albicans*.

To evaluate the ability of the knock-out mutants to grow under oxidative stress, we tested the mutants on YPD media supplemented with different concentrations of hydrogen peroxide and menadione (**Figure 6B**). At 0.15 mM menadione, the mutants *spt7Δ/Δ* showed susceptibility compared to its wild type, while in 7.5 mM H_2_O_2_ SAGA module subunits *spt7Δ/Δ* and *spt8Δ/Δ* showed sensitivity compared to its wild type. This finding suggests that Spt7 and Spt8 might play important roles in mediating oxidative stress resistance.

We examined the response to osmotic stress agents NaCl, CaCl_2_ and glycerol. Interestingly, the *spt7Δ/Δ* and *spt8Δ/Δ* were susceptible to each of 400 mM calcium chloride, 1.5M sodium chloride and 250 mM glycerol compared to the wild type. No phenotypic aberration was seen in the Ngg1 and Ubp8 mutants. Therefore, disruption of structural module and TBP interaction module subunits reduces osmotic response in *C. albicans* and suggests that both Spt7 and Spt8 play a key role in maintaining osmotolerance (**Figure 6C**).

Antifungal drugs such as Caspofungin and chemicals such as Congo Red are often used to induce cell wall stress in *C. albicans* (Wiederhold et al., 2005; Eisman et al., 2006). Caspofungin and Congo Red interfere with β-glucan synthase and chitin synthase respectively (Roncero and Durán, 1985; Ghannoum and Rice, 1999). Based on previous descriptions of the *ada2Δ/Δ* and *gcn5Δ/Δ* mutants (Bruno et al., 2006; Chang et al., 2015), we tested our mutants against the cell-wall stressors Caspofungin and Congo Red at different concentrations. The HAT-module *ngg1Δ/Δ* mutant was sensitive to 200 µg/ml Congo Red, similar to the *gcn5Δ/Δ* mutant that also compromised the HAT module. The *spt7Δ/Δ* mutant was highly sensitive to both Caspofungin and Congo Red, while the *spt8Δ/Δ* and *ubp8Δ/Δ* strains showed a WT response (**Figure 6D**).

Several studies describe filamentation as a potential phenotypic alteration in response to DNA damage in *C. albicans* (Bachewich et al., 2005; Reichow et al., 2007; Loll-Krippleber et al., 2014). Since the SAGA knock-out mutants have altered filamentation, we exposed the mutants to genotoxic-stress-causing agents, namely, the alkylating agent methyl methane sulfonate (MMS) and the DNA replication inhibitor hydroxyurea (HU). The *spt7Δ/Δ*, *spt8Δ/Δ*, and upb8Δ/Δ mutants showed sensitivity at a concentration of 0.01% MMS, while the *ngg1Δ/Δ* mutant was comparable to wild type. Rich media containing 15 mM HU showed the *ngg1Δ/Δ* strain to be resistant whereas other SAGA mutants exhibited sensitivity compared to wild type (**Figure 6C**).

We also analyzed mutant strains in spot assays in media supplemented with different antifungal drugs—the ergosterol biosynthesis inhibitor fluconazole, the glucan synthase inhibitor anidulafungin, and the aminoglycoside antibiotic protein translation inhibitor hygromycin B. Spt7p appears to be a crucial component when it comes to response to drug treatments, as the *spt7Δ/Δ* strain showed sensitivity to 10 µg/ml fluconazole, 100 µg/ml hygromycin B and 0.25 µg/ml anidulafungin; followed by *spt8Δ/Δ* that was not sensitive to hygromycin B, and lastly by *ngg1*Δ/Δ that conferred sensitivity to hygromycin B whereas *ubp8Δ/Δ* behaves like WT. It appears that the mechanisms of drug response regulation by the SAGA complex are drug-dependent, modulated by the different modules. Also, *ubp8Δ/Δ* shows resistance in the presence of the antifungal drugs (**Figure 6D**). This indicates that both Ubp8 and Ngg1 could act as potential drug targets, a point recently supported experimentally (Zhu et al., 2021).

We also subjected the knock-out mutants to alkaline pH 8.3 and acidic pH 5.0 conditions (Vylkova et al., 2011). *spt7Δ/Δ* and *spt8Δ/Δ* show phenotypic change indicating that architecture module and TBP interaction unit play a role in regulating acidic/alkaline stresses compared to WT (**Figures 6A, B**). However, *ngg1Δ/Δ* and *ubp8Δ/Δ* showed normal growth comparable to WT. When we subjected mutant strains to 30 mM DTT to generate ER stress through the accumulation of unfolded protein; strains with the *spt7Δ/Δ* mutation showed sensitivity (**Figure 6B**), suggesting that Spt7 is required for resistance to ER stress in *C. albicans*.

### Macrophage Engulfment Assay Shows a Faster Engulfment of Filamentous Strains in *C. albicans*

**Figure 7:**
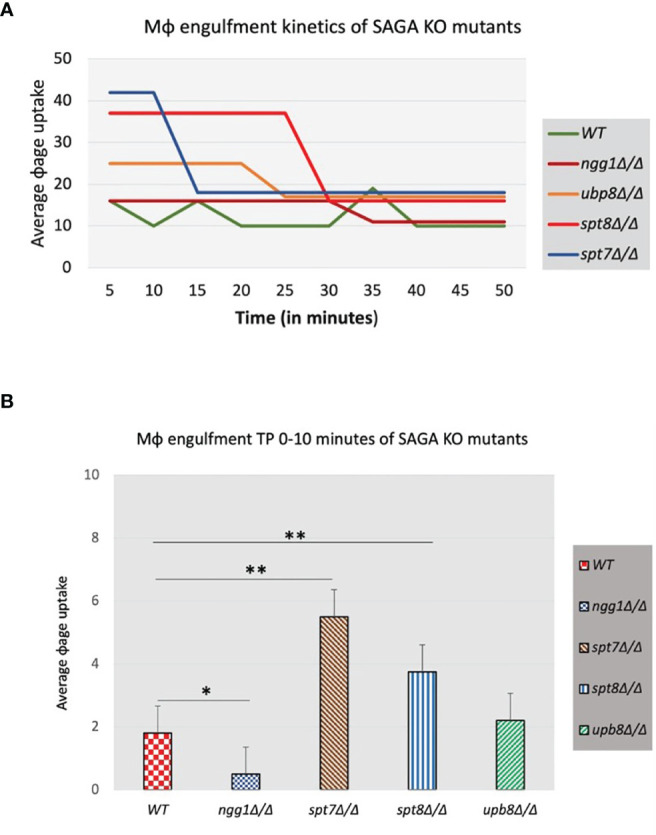
Macrophage engulfment of wildtype and SAGA mutant strains of *C*. *albicans*. Panels **(A, B)** show the time taken for RAW 264.7 murine macrophages to ingest live wildtype and mutant strains following initial cell–cell contact plotted versus the average macrophage uptake. The average time taken for engulfment of the *spt7Δ/Δ* and *spt8Δ/Δ* mutant strains was significantly less while as *ngg1Δ/Δ* was notably slower than for the wildtype control. t-test, **p < 0.01, *p < 0.001. Three biological replicates were made for each mutant. The results from the macrophage engulfment assay suggest that the core structural module subunit Spt7 mutant and the TBP interaction unit component Spt8 mutant could be less virulent, as the constitutively filamentous mutant strains *spt7Δ/Δ* and *spt8Δ/Δ* were quickly recognized and engulfed by macrophages relative to the Ngg1 and Upb8 mutants. Based on the previous evidence, the macrophage engulfment assay supports that SAGA complex sub-modules may work in opposing directions.

Macrophages are a first line of defense against *C. albicans* to prevent the host from developing infections (Lorenz et al., 2004; Krysan et al., 2014). To investigate the function of the SAGA complex in the *C. albicans/*macrophage interaction we tested the knock-out mutants of *ngg1Δ/Δ*, *spt7Δ/Δ*, *spt8Δ/Δ*, and *ubp8Δ/Δ* in a macrophage engulfment assay. We assessed the rate of macrophage engulfment of the different mutants from the SAGA complex which showed that most of the engulfment by macrophages occurred in the first 50 min of interaction between fungal and immune cells (**Figure 7A**), compared to the wild type which showed lower rate of engulfment. This interaction (Candida–macrophage cells) starts at very early timepoints, and the macrophage recognition and further internalization vary among the SAGA mutants. In the first 5 min of interaction, *spt7Δ*/Δ showed a higher rate of engulfment when compared with the wild type (**Figure 7B**), while the *ngg1Δ*/Δ mutant showed a considerably lower rate of engulfment during this period. These results indicate that differences in the cellular morphology might play a role in the variance of macrophage engulfment assays where filamentous strains *spt7Δ*/Δ and *spt8Δ/Δ* were quickly recognized and engulfed by macrophages. However, yeast locked strains *ngg1Δ/Δ* and *ubp8Δ/Δ* showed lower rate of engulfment. This likely explains that SAGA complex subunits might play a role in pathogenicity.

### Expression Analysis of SAGA Module Subunits

As specific null mutants of SAGA complex have a considerable effect on the functioning of *C. albicans*, we performed RNA sequencing for the knock-out strains *ngg1Δ/Δ*, *spt7Δ/Δ*, *spt8Δ/Δ*, and *ubp8Δ/Δ* compared to wild type strain cultured in yeast growth conditions to assess differences in gene expression. Using a statistical-significance analysis with a P-value less than 0.05, we selected the upregulated or downregulated genes with a transcription ratio higher than 1.5-fold change or lower than -0.5-fold change relative to the wild type. We found that in the *spt7Δ/Δ* strain 104 genes were up and 280 genes were downregulated, and in the *spt8Δ/Δ* strain 94 genes were up and 318 genes were downregulated. In *ngg1* mutant strains 138 genes were upregulated and 133 genes were downregulated. *ubp8* null mutants had less of an effect on general gene expression 21 up and 7 genes downregulated (**Tables S3–S6**).

Analysis using the Candida Genome Database GO Term Finder revealed that among upregulated genes in the *spt7* mutant, 42% (44/104 genes) were related to carbohydrate transport (p-value 2.2 × 10^−17^), while in *ngg1* mutants, 19% (26/138) genes were involved in carbohydrate metabolic process, a (p-value 2.5 × 10^−11^); no particular functional class was dramatically enriched in either the *spt8* or the *upb8* mutants. Among the downregulated genes in the *spt7* mutant 11% (36/318) were related to the ergosterol biosynthetic pathway (p-value of 7.3 × 10^−12^), and 13% (39/318) genes were related to carbohydrate metabolism (p-value 4.2 × 10^−11^). In the *spt8* mutant 30% (83/280) of downregulated genes were involved in small molecule metabolic process (p-value of 2.2 × 10^−11^) and 12.5% (35/280) were related to ergosterol biosynthetic pathway (p-value of 1.5 × 10^−12^). SAGA mutants have significant effects on the number of cellular processes, we found that several genes increase or decrease their expression during the yeast-hyphal transition, adhesion, biofilm formation, stress responses, lipid and carbohydrate metabolic processes (**Table 2**).

**Table 2:** Table showing upregulated and downregulated genes in SAGA module subunits from RNASeq analysis data.

Upregulated genes >2 log2FC				
Processes	*spt7 Δ/Δ*	*spt8Δ/Δ*	*ngg1Δ/Δ*	*ubp8Δ/Δ*
Filamentation	*HWP1, HGT12, PGA13*	*HWP1, ALS3, HGC1, HGT12*, *PGA13, UME6*	*–*	*–*
Cell wall Adhesins	*PGA58, PGA13, PGA31*	*ALS3, PGA13*	*ALS2, ALS4, ALS9*, *PGA10, PGA13*	*–*
C/D Box snoRNA genes	*–*	*C2_05600W_A, C2_02080W_A*, *C5_01160C_A, CR_06210C_A*, *C2_10800W_A, C1_12990W_A*, *CR_06220C_A, C1_13000W_A*, *CR_10460W_A, C1_08970W_A*, *C1_08960W_A*	*–*	*–*
**Downregulated genes <−2 log2FC**				
**Processes**	***spt7 Δ/Δ***	***spt8Δ/Δ***	***ngg1Δ/Δ***	***ubp8Δ/Δ***
Ergosterol biosynthesis	*ERG3, ERG251, UPC2*	*–*	*–*	*–*
Sulfur/Methionine biosynthesis	*–*	*MET3, MET4, MET28, SUL2*	*–*	*–*
Carbohydrate metabolism	*MNN22, MNN1, OYE23, GDH2, PFK2, FBA1, TYE7*	*OYE23, OSM2, TYE7, PFK2*	*–*	*–*

As shown in **Table 2**, some specific classes of genes were upregulated in SAGA complex mutants compared to the reference strain SN148 (Crowther et al., 2012). Core filament genes and cell wall adhesion genes include *HWP1*, *HGT12*, *PGA10*, *PGA13*, *PGA31*, *PGA58*, *ALS2*, *ALS3*, *ALS4*, and *ALS9*. Hwp1 was highly upregulated in *spt7* and *spt8* mutants; this protein is important for adhesion to host cells, hyphal development, biofilm formation and virulence (Bruno et al., 2006; Sellam et al., 2009; Orsi et al., 2014), consistent with the filamentous phenotype of the *spt7* and *spt8* mutants. Pga13, a key player in *C. albicans* morphogenesis and virulence (Gelis et al., 2012) was also upregulated in *spt7* and *spt8* mutants, as was Hgt12, required for expression of glucose transporter genes and induced hyphal growth (Biswas et al., 2007). Furthermore, Hgc1 (Hypha-specific G1 cyclin-related protein) was 4.5-fold upregulated in the *spt8* mutant; it plays a key role in hyphal development (Zheng et al., 2004; Buckley, 2008). A second class consists of genes encoding transcription factors that positively regulate hyphal development and enhance biofilm formation *via* Hgc1-Ume6 (Banerjee et al., 2013). Ume6, a true hyphae transcription factor (Banerjee et al., 2008; Zeidler et al., 2009) is 2.5-fold upregulated in the *spt8* mutant.

Intriguingly, in *spt8* mutants a group of C/D box type snoRNAs (small nucleolar RNAs) representing about 25% of all C/D box type snoRNAs in *C. albicans*, was upregulated from 2-fold to 6.5-fold. snoRNAs are non-coding RNAs involved in the single nucleotide modifications of other RNAs (Reichow et al., 2007), and are implicated in nucleolytic processing of ribosomal RNA precursors, telomeric DNA synthesis and alternative splicing (Maxwell and Fournier, 1995; Tollervey and Kiss, 1997; Matera et al., 2007). There are two classes of snoRNAs—C/D box type and H/ACA box type—that are distinguished by structure and their involvement in specific chemical modifications (Balakin et al., 1996; Tollervey and Kiss, 1997; Darzacq et al., 2002). It will be interesting to investigate how SAGA complex mutant *spt8* influences expression of a set of box C/D type snoRNAs.

Certain classes of genes were downregulated in SAGA complex mutants compared to the reference strain SN148 (Crowther et al., 2012). A notable class includes the ergosterol biosynthesis elements *ERG3*, *ERG251*, and *UPC2. Erg3* has key role in azole drug resistance (Zhou et al., 2018) and is 2.9-fold down in the *spt7* mutant; *Upc2* is a transcription factor that is central to the regulation of ergosterol biosynthesis (Vasicek et al., 2014) and is 2.8-fold down in the *spt7* mutant and 1.3 fold down in *spt8* mutant; both mutants showed azole sensitivity.

## Discussion

The SAGA complex, and related complexes SLIK and ADA, are well-studied transcriptional regulators in eukaryotic organisms such as *S. cerevisiae*, *Drosophila melanogaster* and humans (Koutelou et al., 2010; Gurskiĭ et al., 2013; Srivastava et al., 2015). While “rewiring” of orthologous transcriptional regulators in different species can activate or repress unrelated biological processes (Whiteway et al., 2015), this rewiring may also occur in general transcriptional regulators. In the SAGA complex Spt3 (TBP binding module) negatively modulates filamentation in *C. albicans*, opposite to its role in *S. cerevisiae*, while Gcn5 (HAT module) influences morphogenesis in a similar manner in both species, suggesting a possible role of rewiring for this co-activator complex (Laprade et al., 2002).

Here we have provided an overview of different components of SAGA complex regulating growth, morphogenesis, invasiveness, biofilm formation and response to environmental stresses in *C. albicans* (**Table 3**). Overall the Spt7 (Core structural module) and Spt8 (TBP binding unit) components of SAGA complex are involved in negative regulation of filamentation, as was previously noted for Spt3 (TBP binding module) (Laprade et al., 2002), while Ngg1 (HAT module) and Ubp8 (DUB module) appear to positively modulate filamentation, as was also found for Gcn5 of the HAT module (Chang et al., 2015). While the wild type can change its morphological state depending on the conditions, none of the SAGA mutants can switch to morphological state upon changing conditions; they are all locked into their respective morphological states (**Figure 1**). Both Spt7 and Spt8 play important roles in aspects of cell division as both showed cytokinesis defects (**Figures 1**, **4**), as previously described for both *Caspt20Δ/Δ* and Sc*spt20Δ/Δ* (Laprade et al., 2002; Desai and Mitchell, 2015). Our results also indicate that architecture module and TBP interaction unit defects influence cell cycle transitions, causing pseudo-hyphal growth and difficulties in cell separation. Although *spt7* and *spt8* mutant cells showed many similarities in their phenotypes, *spt7Δ/Δ* cells were often binucleated whereas *spt8Δ/Δ* were mononucleated (**Figure 4**), suggesting specific differences in cell cycle regulation (Berman, 2006).

**Table 3:** Table showing the overall results of SAGA mutants in different stress conditions.

Type of stress	Method of stress	*ngg1Δ/Δ*	*spt7Δ/Δ*	*spt8Δ/Δ*	*ubp8Δ/Δ*
**Temperature**	**37°C**				
	**42°C**		sensitivity	sensitivity	
**pH**	**pH 5.0**				
	**pH 8.5**				
**Oxidative**	**H_2_O_2_ 7.5mM**		sensitivity	sensitivity	
	**Menadione 0.15 mM**		sensitivity		
**ER stress**	**DTT 30 mM**		sensitivity		
**Osmotic**	**NaCl 1.5 mM**		sensitivity	sensitivity	
	**CaCl_2_ 400 mM**		sensitivity	sensitivity	
	**Glycerol 250 mM**		sensitivity	sensitivity	
**DNA damage**	**MMS 0.01%**			sensitivity	
	**HU 30 mM**		sensitivity		
**Antifungal**	**Fluconazole 10 μg/ml**		sensitivity	sensitivity	
	**Hygromycin B 100 μg/ml**		sensitivity	sensitivity	
	**Anidulafungin 0.25 μg/ml**		sensitivity	sensitivity	
**Cell wall**	**Caspofungin 0.75 μg/ml**		sensitivity	sensitivity	
	**Congo Red 200 μg/ml**		sensitivity	sensitivity	
**Cell morphology**	**Filamentation**	decreased	increased	increased	decreased
	**Growth rate**		slow	slow	fast
	**Invasiveness**		increased	increased	
	**Biofilm**	increased	decreased	increased	increased

The color code shows differences between the growth of different strains with pink depicting sensitivity or decreased growth and green showing increased growth.

SAGA mutants of *C. albicans* have differences in growth rates—with the slow growers *spt7Δ/Δ* and *spt8Δ/Δ*, similar to that of the *S. cerevisiae* mutants *Scspt7Δ*, *Scspt20Δ* (Core structural module) (Gansheroff et al., 1995; Belotserkovskaya et al., 2000; Lee et al., 2000; Wu and Winston, 2002; Wang et al., 2020) and *Scspt3Δ* (TBP binding module) (Laprade et al., 2002)*;* wild type growers like *ngg1Δ/Δ* similar to *gcn5Δ/Δ* (Chang et al., 2015) and an apparently somewhat faster grower, *upb8Δ/Δ* (DUB module). The increased growth in rich medium of *upb8Δ/Δ* suggests that the control of growth rate may represent a balance between the selective advantages of fast growth and the need to maintain the integrity of the genome (Pir et al., 2012).

It appears that the core structural module (Spt7) plays a role in biofilm formation, while the TBP binding module (Spt8), HAT module (Ngg1), and DUB module (Ubp8) components seem to act as repressors of biofilm formation (**Figure 5**).

We tested the sensitivity of SAGA mutants for different stress conditions. Similar to the baker’s yeast, Ngg1 from the HAT module positively regulates transcriptional response to cell wall perturbations and negatively regulates response to heat stress (Piña et al., 1993). However, it also negatively regulates response to DNA damage stress, another example of rewired regulatory circuitry during the evolution of these fungi. Intriguingly, *gcn5Δ/Δ* mutants of the same module are described to be normal in responding to H_2_O_2_, CPT, MMS, and HU.

In *S. cerevisiae*, the HOG1 pathway controls the synthesis and storage of glycerol which increases intracellular osmotic/turgor pressure to allow cells adapt to high osmotic environments (Saito and Tatebayashi, 2004). Mutations that affect SAGA structural integrity play a critical role for survival at high osmolarity (Daniel and Grant, 2007): the HOG1 MAP kinase pathway recruits SAGA and promotes RNA polymerase II binding to initiate transcription (Proft and Struhl, 2002; Wang et al., 2020).

Antifungal drugs such as Caspofungin and chemicals such as Congo red are often used to induce cell wall stress in *C. albicans* (Wiederhold et al., 2005; Eisman et al., 2006; Plaine et al., 2008). Caspofungin and Congo red interfere with β-glucan synthase and chitin synthase respectively. *Spt7Δ/Δ* and s*pt8Δ/Δ* mutants showed hypersensitivity to Congo red and Caspofungin, (**Figure 6D**) comparable to *Scspt20Δ/Δ* and *Caspt20Δ/Δ* (Lesage et al., 2004; Desai and Mitchell, 2015). Our findings also showed *spt7Δ/Δ* and *spt8Δ/Δ* mutant strains are susceptible to the antifungal agents hygromycin B and anidulafungin B, and to azoles particularly fluconazole (**Figure 6D**) which is consistent with our RNASeq data that showed decreased expression of ergosterol biosynthetic genes (Borecká-Melkusová et al., 2009). Our results also revealed that HAT module (*ngg1*Δ/Δ) and DUB module (*ubp8Δ/Δ*) mutants are resistant to azoles and might act as potential drug targets in *C. albicans* (Zhu et al., 2021).

Macrophages form the first line of immune response in host against developing *Candida* infections (Lorenz et al., 2004; Krysan et al., 2014). SAGA mutants showed differences in their cellular morphology phenotype that likely explains the variance in the macrophage engulfment kinetics. *spt7Δ/Δ* and *spt8Δ/Δ* strains are filamentous and are quickly recognized by macrophages and engulfed, while yeast-locked *ngg1Δ/Δ* cells were poorly engulfed (**Figure 7B**); it is likely that macrophage engulfment of fungal cells is dependent on type and size of hyphae (Lewis et al., 2012)*. In vivo* assays have shown that various SAGA complex modules play roles in virulence; mutations in TBP interaction module subunit-*spt3Δ/Δ* and HAT module subunit-*gcn5Δ/Δ* have shown avirulent behavior, whereas HAT module subunits-*ada2Δ/Δ*, *ngg1Δ/Δ*; TBP interaction unit component-*spt20Δ/Δ* and DUB module subunits—*ubp8Δ/Δ*, *sus1Δ/Δ* caused attenuated virulence in mouse infection models (Laprade et al., 2002; Sellam et al., 2009; Chang et al., 2015; Desai and Mitchell, 2015; Xiao et al., 2018; Shivarathri et al., 2019; Zhu et al., 2021), and the *ngg1* mutant phenotype has been corroborated (Li et al., 2017).

Overall, we found that the single subunit of the recruitment module, Tra1, appeared essential as we failed to obtain a homozygous deletion of *TRA1*. We also found that both the core structural module subunit Spt7 and TBP interaction subunit Spt8 act as repressors of filamentation and invasiveness whereas HAT module subunit Ngg1 and DUB module subunit Ubp8 act as positive regulators. We have shown that both Spt7 and Spt8 play important roles in maintaining SAGA integrity and are critical for normal growth in *C. albicans*. Both *spt7* and *spt8* mutants have shown cell cycle related defects as mother and daughter cells fail to separate during cytokinesis (**Figures 1**, **4**). As well, we have shown that Spt7 and Spt8 are critical for normal *C. albicans* response to DNA damage stress and thus play key roles in maintaining genome integrity, and that Spt7 and Spt8 are vital for the normal response of cells to cell wall, heat, ER, and alkaline stress. Our study also reveals that filamentous strains *spt7Δ/Δ* and *spt8Δ/Δ* are quickly engulfed by macrophages which indicate that SAGA might play a role in pathogenicity in *C. albicans*. All these outcomes imply that core structural module and TBP interaction unit are indispensable for cell morphology, genotoxic and cellular stress responses whereas HAT module plays a role in cell wall stress response. Also, from our findings it appears that HAT module Ngg1 and DUB module Ubp8 might serve as potential drug targets in *C. albicans*.

## Data Availability Statement

The datasets generated during and/or analyzed during current study have been deposited in NCBI’s Gene Expression Omnibus and are accessible through GEO Series accession number GSE193907 https://www.ncbi.nlm.nih.gov/geo/query/acc.cgi?acc=GSE193907.

## Author Contributions

SR and MW were responsible for conceptualization of study. SR, TC-M and MW were responsible for the study design. TC-M-GRACE library screening and methodology, SR- Growth analysis, Invasion assays, Biofilm formation assays, Sensitivity assays and Microscopy. SR and TC-M did the computational pipeline and statistical analysis. RP-RNA isolation, PG- macrophage engulfment assay, SR- RNASeq analysis, SR and TC-M writing original draft preparation. MW-supervision, editing and review. All authors listed have made a substantial, direct, and intellectual contribution to the work and approved it for publication.

## Funding

This work was supported by CIHR MOP42516, CRC 950-228957, and NSERC discovery grant RGPIN/4799.

## Conflict of Interest

The authors declare that the research was conducted in the absence of any commercial or financial relationships that could be construed as a potential conflict of interest.

## Publisher’s Note

All claims expressed in this article are solely those of the authors and do not necessarily represent those of their affiliated organizations, or those of the publisher, the editors and the reviewers. Any product that may be evaluated in this article, or claim that may be made by its manufacturer, is not guaranteed or endorsed by the publisher.
