# Supplementary material for: SAGA Complex Subunits in Candida albicans Differentially Regulate Filamentation, Invasiveness, and Biofilm Formation
Source: Front Cell Infect Microbiol. 2022 Mar 8;12:764711. doi: 10.3389/fcimb.2022.764711 (PMC8957876; doi:10.3389/fcimb.2022.764711)
Supplement: Supplementary file 1 [file DataSheet_1.docx]

**Table S1.** *Strains used in this study.*

| *Strains* | *Parental* | *Genotypes* | *Reference* |
| --- | --- | --- | --- |
| *CASS1* | *CAI4* | *his3::hisG/his3::hisG leu2::tetRGAL4AD-URA3/LEU2* | *Roemer et al. (2003)* |
| *NGG1tetR* | *CASS1* | *ngg1::his3::hisG/his3::hisG leu2::tetRGAL4AD-URA3/LEU2* | *Roemer et al. (2003)* |
| *TRA1tetR* | *CASS1* | *tra1::his3::hisG/his3::hisG leu2::tetRGAL4AD-URA3/LEU2* | *Roemer et al. (2003)* |
| *SPT7tetR* | *CASS1* | *spt7::his3::hisG/his3::hisG leu2::tetRGAL4AD-URA3/LEU2* | *Roemer et al. (2003)* |
| *SPT8tetR* | *CASS1* | *spt8::his3::hisG/his3::hisG leu2::tetRGAL4AD-URA3/LEU2* | *Roemer et al. (2003)* |
| SN148 | SN76 | *arg4/arg4  leu2/leu2   his1/his1*  *ura3::imm434/ura3::imm434*  *iro1::imm434/iro1::imm434* | Noble & Johnson (2005) |
| *ngg1∆/∆* | SN148 | ngg1::NGG1*/ngg1::NGG1* (CRISPR/CAS9) *arg4/arg4 leu2/leu2 his1/his1* *ura3::imm434/ura3::imm434* *iro1::imm434/iro1::imm434* | This study |
| *spt7∆/∆* | SN148 | spt7::SPT7*/spt7::SPT7* (CRISPR/CAS9) *arg4/arg4 leu2/leu2 his1/his1* *ura3::imm434/ura3::imm434* *iro1::imm434/iro1::imm434* | This study |
| *spt8∆/∆* | SN148 | spt8::SPT8*/spt8::SPT8* (CRISPR/CAS9) *arg4/arg4 leu2/leu2 his1/his1* *ura3::imm434/ura3::imm434* *iro1::imm434/iro1::imm434* | This study |
| *ubp8∆/∆* | SN148 | ubp8::URA3/ubp8::URA3 (CRISPR/CAS9) *arg4/arg4 leu2/leu2 his1/his1* *iro1::imm434/iro1::imm434* | This study |

*Insertion of 3 stop codons in the repair DNA

**Table S2.** *Primers used in this study.*

| **Name** | **Sequence (5′ to 3′)** |
| --- | --- |
| NGG1_sgRNA_F | atttgAGAATTAACACCAGAACACCg |
| NGG1_sgRNA_R | aaaacGGTGTTCTGGTGTTAATTCTc |
| NGG1_HR_F | ATTCAAAAACTTCCGAAAACGATAAAAAACGTAAAAATGAAGAATT CACATGATAACACC |
| NGG1_HR_R | TTCTTTGTTGCGCTCATAGGCACTTCGTCTTCATCGTCATGGTGTTATCA TGTGAATTCT |
| NGG1_Ex_F | GACTGATGCGCACTCTGTGTC |
| NGG1_Ex_R | CTCTTCCGACCAAAGATCCGC |
| SPT8_sgRNA_F | atttgAAATGAAGACGAGGAAGGTGg |
| SPT8_sgRNA_R | aaaacCACCTTCCTCGTCTTCATTTc |
| SPT8_HR_F | GGCGATGAAGATGAAGAAATGGCAGATGAAGATGGCGCATATGAAG ACTAGTAAGGTG |
| SPT8_HR_R | GCTCGTATCTTCTTCATCTTCTTCTTCTTCTTCTTACTCACCTTACTAGTCT TCATAT |
| SPT8_Ex_F | CATCAATCGAACAAGACGATC |
| SPT8_Ex_R | GTTAATGGTTGTTCATTTTCC |
| SPT7_sgRNA_F | atttgAATGAGAACAACGAGAGTGCg |
| SPT7_sgRNA_R | aaaacGCACTCTCGTTGTTCTCATTc |
| SPT7_HR_F | CCCGAAGATAAGAATGACAAACCTGAGACTCTAGACACCAATGAGAAC TAATAGAGTGC |
| SPT7_HR_R | ATCTCTATCACTACTATTGTTCTGAATTCTCTCACCAACAGCACTCTCGTTG TTCTCATT |
| SPT7_Ex_F | GCGATCTCTATGAAAAGCAAC |
| SPT7_Ex_R | CATCTTCATCTTCGTCCTCG |
| UBP8_sgRNA_F | atttgTGCCACCAATAATATCAATg |
| UBP8_sgRNA_R | aaaacCATTGATATTATTGGTGGCa |
| UBP8_HR_URA3_F | taatatataaATGCCTTCTGATGAAACAATATCTAAATAAAATGGCAATATCCACA TTGCCACGAATTCTATCTAATGAGGTTTATATACCGCCCCTTTT |
| UBP8_HR_URA3_R | CTCGTACAAAATTTATTTTATTATGCAATAATGTTGATGATTGATCAGAAAT GTCTAGTTGATCTGTAGTAGTAGGACCACCTTTGATTGTAAATAGT |
| UBP8_Ex_F | AACATCCATCTTCTCCTTGGCA |
| UBP8_Ex_R | CTTCTCCTCGTCGTGTTCACCT |
| URA3-F | TTGGGCAGATATTACCAATGC |
| URA3-R | GCTAAAGAAACCACCACCAA |
